# Fusion of a Tooth with a Supernumerary Tooth: A Case Report and Literature Review of 35 Cases

**DOI:** 10.3390/children11010006

**Published:** 2023-12-20

**Authors:** Tatsuya Akitomo, Satoru Kusaka, Momoko Usuda, Mariko Kametani, Ami Kaneki, Taku Nishimura, Masashi Ogawa, Chieko Mitsuhata, Ryota Nomura

**Affiliations:** Department of Pediatric Dentistry, Graduate School of Biomedical and Health Sciences, Hiroshima University, 1-2-3 Kasumi, Minami-ku, Hiroshima 734-8553, Japan; higechi@hiroshima-u.ac.jp (S.K.); musuda@hiroshima-u.ac.jp (M.U.); mrysk25@hiroshima-u.ac.jp (M.K.); kaneki@hiroshima-u.ac.jp (A.K.); nishi04@hiroshima-u.ac.jp (T.N.); caries0@hiroshima-u.ac.jp (M.O.); chiekom@hiroshima-u.ac.jp (C.M.); rnomura@hiroshima-u.ac.jp (R.N.)

**Keywords:** fused tooth, supernumerary tooth, case report, literature review

## Abstract

Tooth fusion is a dental anomaly with a prevalence of 0.1% in permanent dentition. The aim of this paper is to present the occurrence, region of fusion, and prognosis of fused tooth which is a very rare case in the literature. The authors present a very rare case with a maxillary right lateral incisor fused to a supernumerary tooth on the palatal side. The eruption was delayed, but eruption occurred naturally at 9 years and 6 months. However, cone-beam computed tomography at 11 years and 6 months revealed it was diagnosed as a fused tooth. The authors undertook a literature review over the past 10 years and found 30 articles about fused teeth between permanent teeth and supernumerary teeth. It was more common in males than females and there were no differences depending on the occurrence. Mesiodistal fusion was more common in the incisor region, while labiopalatal fusion was more common in the molar region. Most cases required dental treatment with a surgical approach, root canal treatment, or orthodontic treatment. The present study highlights the importance of long-term follow-up and suggests the region of fusion and prognosis of the fused tooth may vary depending on the occurrence.

## 1. Introduction

Dental anomalies include abnormal tooth counts, morphology, size, and eruption times [1,2,3,4]. Congenital types are inherited and have a genetic basis, developmental types occur at the tooth formation stage, and acquired anomalies occur after tooth development [5]. Anomalies in number, morphology, and size are included in developmental types, and a clinician may encounter in pediatric dentistry [5,6]. Dental anomalies arise from the interaction of genetic, epigenetic, and environmental factors in the process of dental formation [5,7,8]. These can lead to functional, occlusal, and aesthetic problems due also to alterations to the process of dental eruption, so early intervention is crucial under certain conditions [8].

Double teeth are dental anomalies concerning tooth morphology and are classified into fusion and gemination, which are similar anomalies of shape that have different etiologies [9,10]. Fusion is the union of one or more teeth during development, while gemination occurs when two separate morphological units are created by the division of the tooth germ [11]. The prevalence of these anomalies varies, with the prevalence reported to be higher in Japan than in Western countries [12,13].

A supernumerary tooth fused to a normal tooth often causes malocclusion and esthetic problems because of its abnormal shape and large crown and requires treatment such as sectioning and extraction of the supernumerary tooth, extraction, orthodontic treatment, or root canal endodontic treatment [14,15,16]. Although there are many case reports in which fused teeth are detected and treated, few of these cases are detected during follow-up over the long term. In addition, few reports have described the occurrence, region of fusion, and prognosis of fused teeth.

The authors encountered a rare case of a child with a maxillary right lateral incisor fused to a supernumerary tooth on the palatal side with long-term follow-up. Herein, the authors present the case report and a literature review on fused teeth over the past 10 years. The aim of this study is to present the occurrence, region of fusion, and prognosis of fused teeth which is a very rare case in the literature.

## 2. Case Presentation

**Figure 1:**
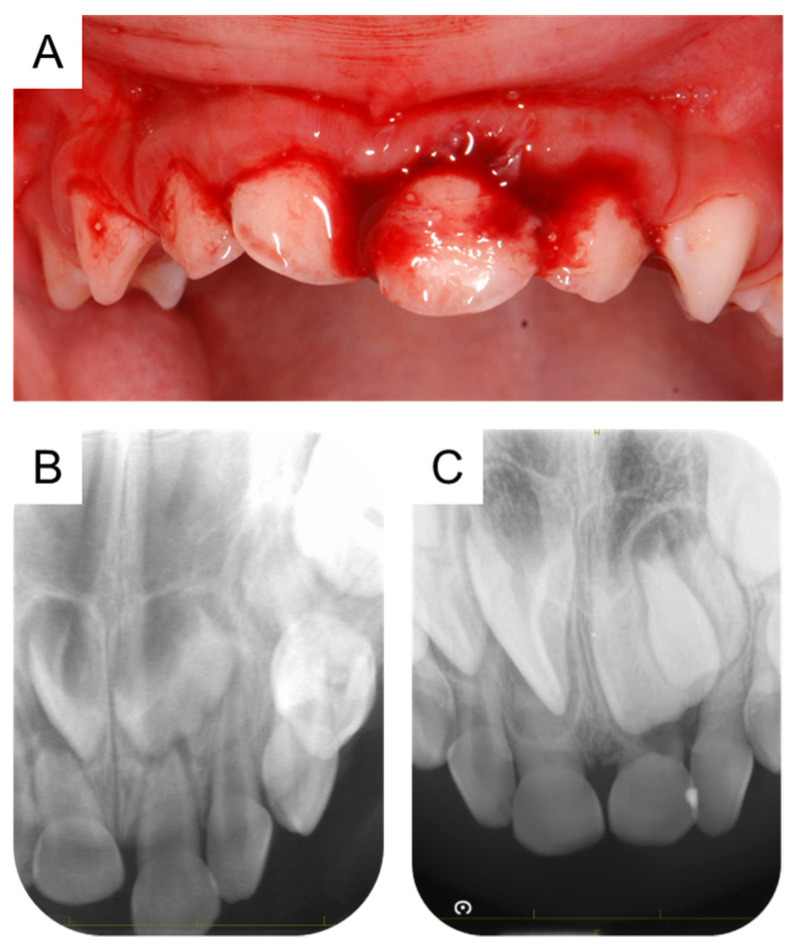
Intraoral photograph and periapical radiographs before the eruption of the maxillary permanent incisors. (**A**,**B**) Initial examination at the age of 2 years and 1 month. (**C**) Periapical radiograph at the age of 6 years and 5 months.

A 2-year and 1-month-old Japanese boy was referred to the Pediatric Dentistry Clinic of Hiroshima University Hospital with the chief complaint of dental trauma to the maxillary primary teeth. The medical history revealed that the patient had attended the Pediatric Clinic of Hiroshima University Hospital with atopic dermatitis. The patient’s elder sister had a congenital absence of the mandibular bilateral central incisors and the left lateral incisor, and his younger brother had a congenitally absent mandibular right central incisor. Intraoral examination showed palatal displacement of the maxillary right primary central incisor and extrusion of the maxillary left primary central incisor (Figure 1A). No tooth fracture was observed on the periapical radiograph (Figure 1B). The traumatized tooth was fixed in place for 1 month, and the authors continued the follow-up thereafter. Although pulp cavity stenosis and external root resorption were observed at the age of 6 years and 5 months, there were no other abnormal findings. Therefore, the authors continued to follow up (Figure 1C). The central incisors were replaced by permanent teeth at the age of 7 years and 10 months.

**Figure 2:**
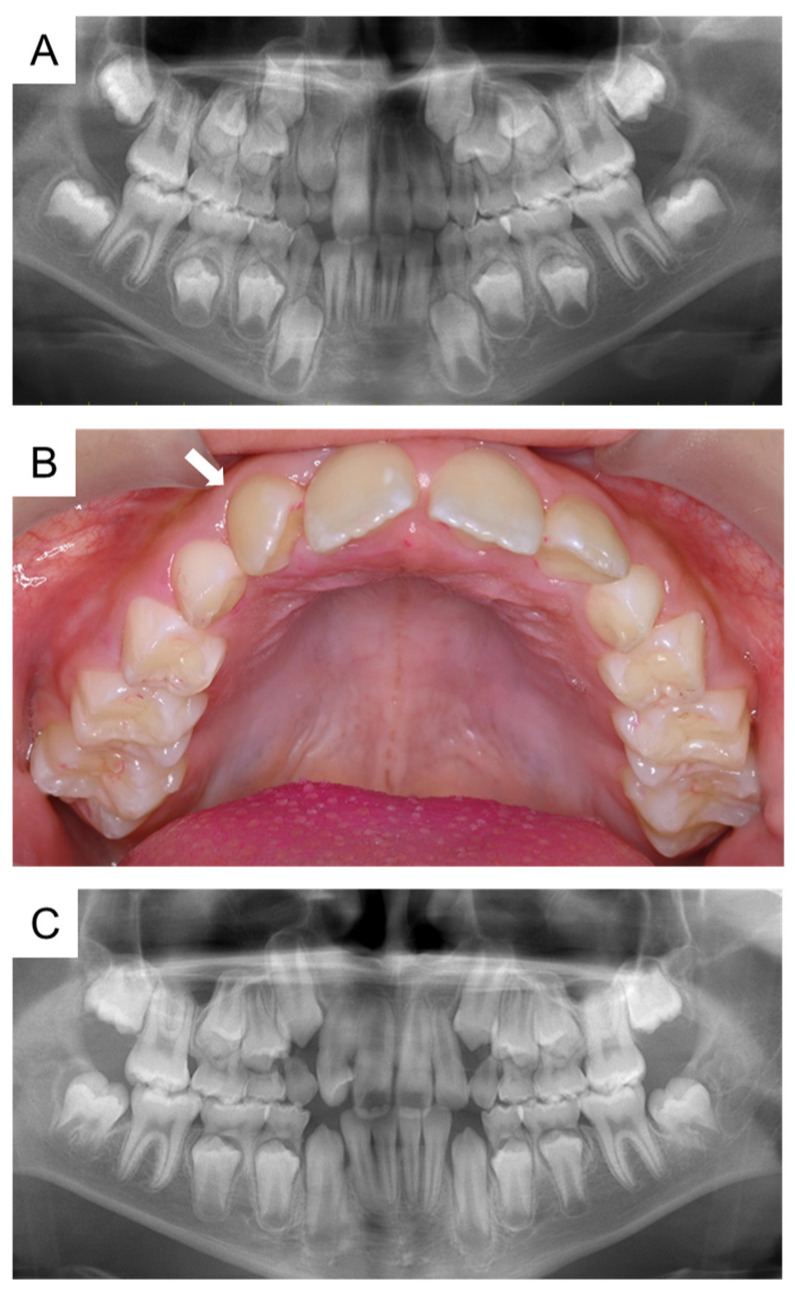
Panoramic radiographs and intraoral examination radiographs before and after the eruption of the maxillary permanent incisors. (**A**) Panoramic radiograph at the age of 8 years and 10 months. (**B**) Intraoral photograph at the age of 10 years and 6 months. Arrows indicate the maxillary right lateral incisor. (**C**) Panoramic radiograph at the age of 10 years and 10 months.

**Figure 3:**
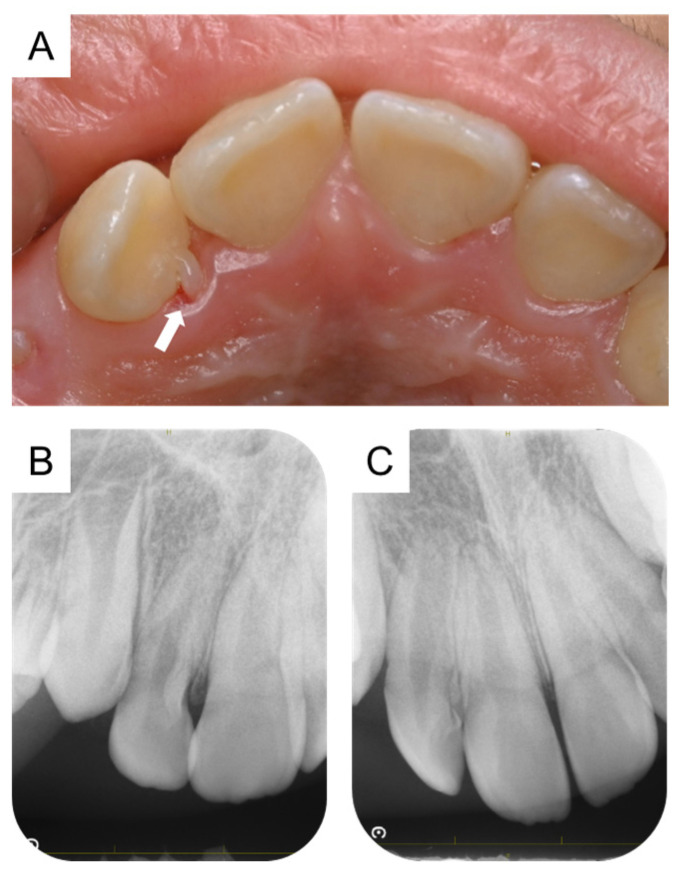
Intraoral photograph and periapical radiograph of maxillary right lateral incisor showing calcified tissue resembling a talon cusp at the age of 11 years and 6 months. (**A**) Intraoral photograph. Arrows indicate the calcified tissue similar to a talon cusp on the palatal side of the maxillary right lateral incisor. (**B**,**C**) Periapical radiographs.

At the age of 8 years and 10 months, a panoramic radiograph revealed that the maxillary right lateral incisor was slightly twisted in the alveolar bone, and eruption was delayed compared with the left side (Figure 2A). A follow-up observation confirmed the natural eruption of the maxillary right lateral incisor at the age of 9 years and 6 months. Although there was no abnormal finding at the intraoral examination at the age of 10 years and 6 months, a panoramic radiograph at the age of 10 years and 10 months revealed a radiolucent line on the palatal side of the maxillary right lateral incisor (Figure 2B,C). At the age of 11 years and 3 months, the authors detected calcified tissue similar to a talon cusp. Three months later, the calcified tissue had become more obvious, and a periapical radiograph was taken (Figure 3A,B). However, it was difficult to make a definitive diagnosis, and the cone-beam computed tomography (CBCT) images were taken for a more detailed examination.

**Figure 4:**
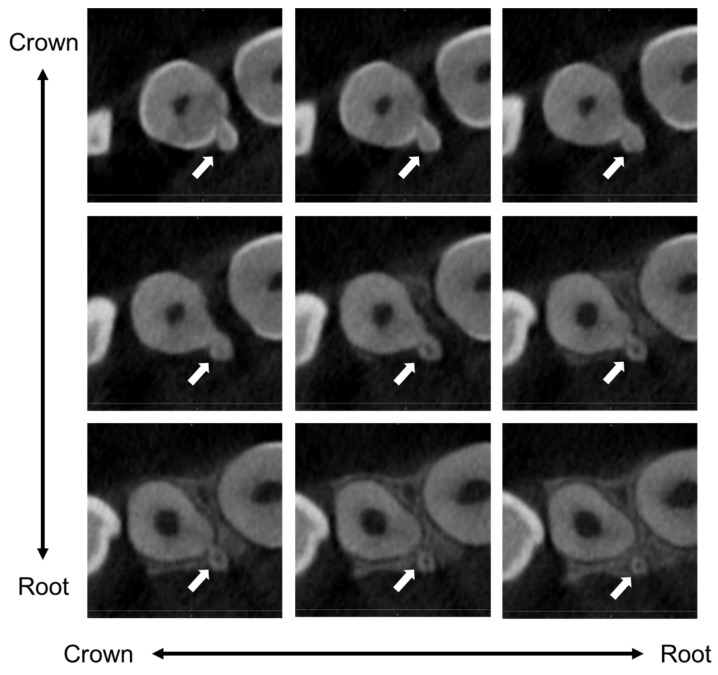
Cone-beam computed tomography imaging of a horizontal section at the age of 11 years and 6 months. The calcified tissue had a crown, root, and pulp chamber; was independent of the maxillary right lateral incisor; and was diagnosed as a supernumerary tooth. Arrows indicate the tooth fused with the supernumerary tooth.

**Figure 5:**
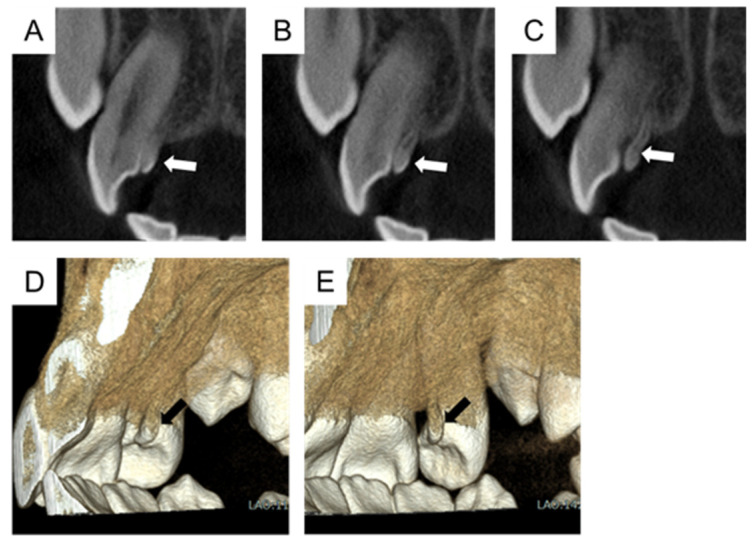
Cone-beam computed tomography imaging of the sagittal section and three-dimensional construction images at the age of 11 years and 6 months. (**A**–**C**) The sagittal section. (**D**,**E**) Three-dimensional construction images. Arrows indicate the fused tooth with the supernumerary tooth.

The CBCT instrument was 3D Accuitomo F17 (Morita Corp, Kyoto, Japan), and the scanning protocol involved a field of view of 40 × 40 mm, a multi-planar reformation of 1 mm thickness, a tube voltage of 90 kV, and a tube current of 6.5 mA. It revealed the calcified tissue had a crown, root, and pulp chamber independent of the maxillary right lateral incisor, which was diagnosed as a supernumerary tooth (Figure 4 and Figure 5). The supernumerary tooth had continuous enamel and dentin on the palatal side of the maxillary right lateral incisor and was diagnosed as a fused tooth. There were no symptoms or esthetic or functional disorders in the tooth. Therefore, the authors plan to continue the follow-up, including the prevention of dental caries in the fused region.

## 3. Methods

### 3.1. Study Selection

The review protocol is reported in line with the Preferred Reporting Items for Systematic Reviews and Meta-Analyses (PRISMA) statement [17] (see Supplementary File S1). A literature review of PubMed, the electronic database which is provided by the National Library of Medicine via the Internet (https://pubmed.ncbi.nlm.nih.gov/ (accessed on 9 November 2023)) was conducted by one of the authors on 9 November 2023. The articles were searched using the terms “fused tooth” and “supernumerary tooth” and published from 2013 to 2023. Articles that could not be viewed entirely or that were not about human case reports were excluded from this study. This study also searched for articles about the fusion between permanent teeth and supernumerary teeth. Therefore, articles about fusion between permanent teeth were also excluded. If it was unclear which tooth was the supernumerary tooth, the tooth that received conservative treatment was considered the permanent tooth.

### 3.2. Data Extraction

The authors extracted data from articles about fused teeth (objective, sex, tooth number, region of fusion, prognosis, and conclusion). In cases in which it was unknown which tooth was supernumerary or in cases in which not all items were documented, only the items that could be diagnosed from the article were extracted. Prognosis was divided into “Root canal treatment”, “Partial extraction”; sectioning and extraction of supernumerary tooth, “Extraction”; extraction of fused tooth, “Orthodontic treatment” and others were included in “Others”.

## 4. Results

There were 66 articles found from the PubMed search in the survey period. Of the 66 articles, 36 articles were excluded, and 30 articles (35 cases) were extracted that fulfilled the selected criteria. Two fused teeth were present in only one patient. Detailed case information is listed in Table 1 [18,19,20,21,22,23,24,25,26,27,28,29,30,31,32,33,34,35,36,37,38,39,40,41,42,43,44,45,46,47]. The authors reviewed the 36 cases of fused teeth including the case discussed above. There were 23 males and 13 females, with a male-to-female ratio of 1.8:1. Of the total of 37 fused teeth in 36 cases, 28 occurred in the maxilla, 9 in the mandible, 19 occurred on the right side, and 18 on the left side. Twenty-seven fused teeth (73.0%) were present in the incisor region, followed by nine fused teeth (24.3%) in the molar region, and one fused tooth (2.7%) in the premolar region. There were no fused teeth in the canine region.

Of the 27 fused teeth in 26 cases that occurred in the incisor region, there were 16 males and 10 females, with a male-to-female ratio of 1.6:1. Most of the fused teeth in the incisor region were in the maxillary central incisor region (19 teeth; 70.4%), followed by 5 (18.5%) in maxillary lateral incisor region, and 3 (11.1%) in the mandibular lateral incisor region. No fused teeth were reported in the mandibular central incisor region. There was no difference in incidence between the left and right sides. Twenty-five teeth were fused mesiodistally, and two teeth were fused labiopalatally. Eleven teeth (40.7%) underwent a surgical approach such as partial extraction (nine teeth) or extraction of the fused tooth (two teeth). Of the nine cases in which partial extraction was performed, three cases each received root canal treatment or orthodontic treatment, including duplicates. Even if a surgical approach was not used, most of the fused teeth had undergone root canal treatment or orthodontic treatment.

Of the 10 fused teeth occurring in the molar region, 7 were male and 3 were female, with a male-to-female ratio of 2.3:1. Seven fused teeth (70.0%) involved the second molar, followed by two fused teeth (20.0%) in the third molar region and only one tooth (10.0%) in the second premolar region. However, the location of occurrence (maxilla or mandible, right or left) was irregular. Nine teeth were fused labiopalatally and only one tooth was fused mesiodistally. Seven teeth, including one partial extraction case, had undergone root canal treatment, one tooth had been extracted, and two had undergone other treatment. Most of the reports were concluded to highlight the usefulness of CBCT and the importance of a multidisciplinary approach to its management.

## 5. Discussion

### 5.1. Fused Tooth

The etiology of the fusion of teeth is unknown; however, it is believed that it involves physical force or pressure from the follicles of adjacent teeth, hereditary conditions, and racial determinants [48]. Duncan et al. (1987) reported that the prevalence of unilateral double teeth was 0.5% in the primary dentition and 0.1% in the permanent dentition, indicating that it is more common in the primary dentition than in the permanent dentition [48,49]. As mentioned above, double teeth are classified into fusion and gemination; however, the differential diagnosis between fusion and gemination is difficult only when there is a fusion between a normal tooth and a supernumerary tooth [11,49]. The fused tooth is very rare, and few reports have described the occurrence, region of fusion, and prognosis. The objective of this study is to review literature over the past 10 years and present them.

### 5.2. The Present Case

In this case, fused teeth were observed in permanent teeth, which is generally rare. Fused teeth often appear mesiodistally in the incisors, and the teeth may be partially or completely extracted for aesthetic restoration. In the present case, the fused tooth had a supernumerary tooth on the palatal side. Therefore, few aesthetic problems were caused, and a surgical approach was not necessary. Furthermore, most fused teeth are discovered immediately before or after the eruption. In contrast, the fused teeth in our case were discovered during long-term follow-up observation because they were in an anatomical position that is difficult to detect.

In the present case, no tooth abnormality was observed in the panoramic radiograph at the age of 8 years and 10 months. The diagnosis was difficult from a panoramic examination at that point because the region of fusion was located on the palatal side. The maxillary right lateral incisor fused with a supernumerary tooth erupted naturally at the age of 9 years and 6 months, although its eruption was slightly delayed compared with the opposite side. However, no evidence of fused teeth was found at this time, and calcified tissue, like a talon cusp, was identified approximately 2 years later at an intraoral examination at the age of 11 years and 3 months. Additionally, a CBCT image revealed the presence of an independent tooth crown, pulp chamber, and root within the calcified tissue 3 months later. The supernumerary tooth fused with the lateral incisor had an independent root and the fused tooth which has multiple roots such as premolar, therefore, it was possible that the morphological abnormality affected the difference in the timing of eruption.

### 5.3. Literature Review

The authors investigated the literature published in PubMed about fused teeth within the past 10 years and found 30 articles (35 cases) from 66 articles. Fused teeth were more common in males than in females with a male-to-female ratio of 1.8:1. The same tendency was observed when cases were divided according to the incisor or molar region. Açıkel et al. (2018) investigated the distribution of primary fused teeth and reported 40 cases (24 male and 16 female) out of 13,450 pediatric patients [48]. Although the incidence differs between primary teeth and permanent teeth, it is suggested that it may be more common in males. Basalamah et al. (2016) examined 1000 school children aged 4–12 years looking for oral anomalies and concluded that the total prevalence of oral anomalies was 15.1%, occurring most commonly in boys (male: female ratio 3.2:1) aged 7–12 years [50]. It is possible that not only fused teeth but also other dental anomalies are more common in males.

### 5.4. Occurrence and Region of Fusion

The present study showed that most fused teeth occurred in the incisor region. There were many more reports of affected central incisors than of lateral incisors in the incisor region, whereas, in the molar region, there were many reports of second molars and no reports of first molars. Mesiodistal fusion was most common in the incisor region. Although 25 cases had mesiodistal fusion, only 2 cases of labiopalatal fusion, including this case, were detected. However, labiopalatal fusion was more common in the molar region. Differences in the occurrence or fused region may be related to the tooth formation process.

### 5.5. Prognosis

In the incisor region, the fused tooth was often partially or completely extracted, whereas in the molar region, a conservative approach such as root canal treatment was most frequently adopted. As mentioned above, mesiodistal fusion, which compromises esthetics, is common in the incisor region. Therefore, many of these cases required a surgical approach such as partial or complete tooth extraction. Smail-Faugeron et al. (2016) investigated the systematic review of 101 double teeth occurring in the permanent maxillary central incisor and 40 percent of double teeth required a surgical approach, while there was only 17 percent that did not need treatment [37]. These results suggest that the surgical approach is common in the fused tooth in the incisor region.

On the other hand, most cases in the molar region underwent root canal treatment. Since teeth are essential not only for nutritional intake, it has also been reported by Kondo et al. (2016) that molar loss impaired neurogenesis in the hippocampal dentate gyrus and learning ability in animal experiments [51]. The belief that molar loss affects brain function may have resulted in conservative treatment being chosen rather than tooth extraction as the first choice. This literature review focuses on case reports. Therefore, there were only 35 case reports about fused teeth between permanent teeth and supernumerary teeth for 10 years, and few reports of fused teeth that did not undergo dental treatment. This is a limitation in this report and there may be an increased risk of bias. In future research, the authors would like to consider large-scale surveys of fused teeth, including those that remained untreated.

Cunha et al. (2015) described a case in which a tooth was fused on the palatal side of a maxillary lateral incisor, similar to this case. The patient had spontaneous pain, resulting in root canal treatment of the supernumerary tooth only [27]. In the present case, there have been no symptoms in the fused tooth. Additionally, the patient does not wish to undergo dental treatment such as partial tooth extraction or root canal treatment. The authors continue to maintain long-term follow-up, including radiographic examination. In addition, most of the previous reports highlighted the importance of a multidisciplinary approach facilitate to the successful treatment of this dental abnormality [21,29,31,33,37,38,44,45]. The authors make a plan to cooperate with specialists, such as orthodontics, periodontics, endodontics, and prosthodontics, in multiple fields the in future if necessary.

Finkelstein et al. (2015), who reported four cases of fused teeth, concluded that early diagnosis and treatment are the keys to the successful correction of fused and geminated teeth in the maxillary anterior region [29]. In the present case, regular dental check-ups since the age of 2 years led to early detection of the fused tooth. The calcified tissue gradually became obvious after eruption and CBCT was diagnosed as a fused tooth. There have been many reports on the effectiveness of CBCT [24,28,30,35,36,39,42,46]. However, in the present case, the tooth eruption was delayed compared with the left side in the alveolar bone before CBCT was taken. Other dental abnormalities such as supernumerary teeth and odontomas can also cause delayed tooth eruption [3,52]. This review reconfirms the importance of long-term follow-up, and the present case report highlights the need for detailed examinations to determine if there is a dental abnormality in cases in which tooth eruption is delayed.

## 6. Conclusions

Teeth fused with supernumerary teeth are rare, and a variety of treatments are available such as orthodontic treatment, root canal treatment, partial extraction, and extraction. When the patient has no oral disease such as dental caries or periodontitis, it is desirable for the patient that invasive treatment is avoided. In such cases, it is important to conduct regular check-ups to avoid problems caused by dental abnormalities. In addition, the region of fusion differs depending on the incisor or molar regions, and this may also affect the choice of treatment. The study highlights the importance of long-term follow-up and detecting the fused tooth in the early stage. Further epidemiological research should clarify the detailed prevalence of fused teeth and elucidate the mechanism of fused teeth development in the future.

## Figures and Tables

**Table 1:** Fused teeth reported in the literature.

Author	Objective	Sex	Tooth Number	Fused Region	Prognosis	Conclusions
Demircioglu Guler D, 2013 [18]	To report multidisciplinary management of a fused maxillary anterior tooth.	Male	11	Mesiodistal	Partial extraction and Ortho	The technique described offers a simple and effective method for restoring a fused tooth that reestablishes function, shape, and esthetics.
Gupta R, 2013 [19]	To describe root canal treatment of a fused carious tooth presenting with apical periodontitis.	Female	37	Labiopalatal	RCT	The fused teeth were endodontically treated and restored using porcelain fused to a metal crown.
Gupta SK, 2013 [20]	To describe a unique case of a double tooth in relation to a mandibular lateral incisor exhibiting the presence of four root canals.	Female	32	Mesiodistal	RCT	The role of conventional radiography and advanced three-dimensional imaging techniques in the better assessment of complex root canal systems and their aid in endodontic management has also been highlighted.
Venugopal S, 2013 [21]	To describe the presence of a concrescence between the mandibular second molar and a supernumerary tooth, with its clinical and radiographic findings	Male	47	Labiopalatal	Partial extraction and RCT	A multidisciplinary approach is required for its management.
Cho KM, 2014 [22]	To present a rare case in which fusion of the maxillary left second premolar and a supernumerary tooth in a 13-year-old girl was diagnosed using cone-beam computed tomography.	Female	25	Labiopalatal	RCT	Fused teeth can be effectively managed by the comprehensive treatment which includes both endodontic and periodontal procedures.
Hattab FN, 2014 [23]	To report the dental abnormalities which create clinical, pathological, and esthetic problems.	Female	11	Mesiodistal	Others	Dental practitioner should be aware of the clinical signs, associated problems, and treatment options for a given case.
Jain P, 2014 (Case 1) [24]	To present two case reports on the endodontic management of two maxillary second molars fused to paramolar tubercles.	Male	27	Labiopalatal	RCT	Use of cone-beam computed tomography provided valuable information about the root/root canal anatomy and the EndoVac irrigation system was useful in cleaning the communications that aided in the successful endodontic management.
Jain P, 2014 (Case 2) [24]		Male	27	Labiopalatal	RCT	
Steinbock N, 2014 [25]	To describe a 10-year outcome of a combined treatment of a fused maxillary incisor by means of an orthodontic–endodontic–prosthodontic–oral surgery management protocol.	Female	21	Mesiodistal	Partial extraction and Ortho	Proper interdisciplinary treatment planning of complicated cases such as anomalous teeth, which involve fusion to a supernumerary tooth, may lead to minimal invasive conservative procedures that maintain tooth vitality and result in a pleasing esthetic result.
Yagci A, 2014 [26]	To present the successful resolution of a fused maxillary lateral incisor with a supernumerary tooth using endodontic, surgical, restorative, and orthodontic management.	Female	22	Mesiodistal	Partial extraction, RCT, and Ortho	The decision made in extracting or retaining the fused tooth depends on the arch discrepancy and esthetic needs.
Cunha RS, 2015 [27]	To describe a case of unilateral fusion of a supernumerary tooth to a maxillary permanent lateral incisor in which a conservative approach was used to reach a favorable outcome.	Female	12	Labiopalatal	RCT	Clinicians should be aware of possible anatomic variations and that thorough diagnosis by using appropriate technology is crucial to determine the treatment option that will provide the best outcome.
Das S, 2015 [28]	To describe endodontic, surgical, and restorative management of fused and dilacerated maxillary central incisor.	Male	21	Labiopalatal	Partial extraction and RCT	Three-dimensional imaging with cone-beam computed tomography for accurate diagnosis and interdisciplinary treatment planning for correction of esthetics and function is needed for the successful management of malformed teeth.
Finkelstein T, 2015 (Case1) [29]	To evaluate the prevalence of fused/geminated teeth in the maxillary anterior region of orthodontically treated patients and present treatment options and their outcomes.	Male	11	Mesiodistal	Ortho	Esthetic consideration is a determining factor for various treatment alternatives. A multidisciplinary approach is imperative for the successful treatment of these dental abnormalities.
Finkelstein T, 2015 (Case2) [29]		Male	11	Mesiodistal	Ortho	
Finkelstein T, 2015 (Case3) [29]		Male	21	Mesiodistal	Ortho	
Finkelstein T, 2015 (Case4) [29]		Female	11	Mesiodistal	Partial extraction	
Kato H, 2015 [30]	To report a case of a mandibular second molar fused with a paramolar, necessitating dental pulp treatment.	Male	47	Labiopalatal	Others	Evalualuation using cone-beam computed tomography and modeling with a printer markedly facilitated a three-dimensional understanding of the complicated morphology of the region involved.
Khan R, 2015 [31]	To describe an 8-year-old boy presented with a ‘large tooth’ in the upper front region of his jaw.	Male	11	Mesiodistal	Others	A multidisciplinary approach is required to treat such a condition.
Sharma G, 2015 [32]	To report a rare case of dens invaginatus and dens evaginatus on fused permanent maxillary central incisor with supernumerary tooth in a 40-year-old male, and also focuses on the differentiating fusion from gemination and reviews preventive and management strategies for tooth with complex dental anatomy.	Male	21	Mesiodistal	Others	The rarity of all these dental anomalies occurring together on the same tooth makes it a unique case. A detailed and accurate radiographic examination is required for diagnosis and subsequent management.
Zhu M, 2015 [33]	To report endodontic management and the periodontal therapy of a mandibular second molar that appeared to have been fused with a supernumerary tooth and the importance of the use of cone-beam computed tomography as a valuable diagnostic aid in the treatment of such complex cases.	Female	47	Labiopalatal	RCT	A multispecialty approach with dentists of various specialties teaming up for better treatment outcomes can result in a successful culmination of a complicated treatment plan.
Aydemir S, 2016 [34]	To present a rare case of a fused mandibular lateral incisor with a supernumerary tooth with a follow-up for 18 months.	Female	42	Mesiodistal	RCT	Because of the abnormal morphology of the crown and the complexity of the root canal system in fused teeth, treatment protocols require special attention.
Jiang K, 2016 [35]	Based on a case of supernumerary cusp on the bucca of the left maxillary second molar diagnosed by cone-beam computed tomography, its genesis, diagnosis, and antidiastole are to be analyzed.	Male	27	Labiopalatal	Others	Cone-beam computed tomography can improve the accuracy of diagnosis.
Ozcan G, 2016 [36]	To describe the use of cone-beam computed tomography to visualize the fusion of the mandibular third molar and a supernumerary tooth in relation to a paradental cyst and in the presence of a retromolar canal in the same region.	Male	48	Labiopalatal	Extraction	An accurate assessment of morphological and pathological formations was carried out using cone-beam computed tomography.
Smail-Faugeron V, 2016 (Case1) [37]	To report an 11-year-old boy with bilateral fusion of the two maxillary central incisors and a 9-year-old boy with a double left central incisor and a supernumerary lateral right incisor.	Male (2 teeth)	11	Mesiodistal	RCT and Ortho	A multidisciplinary approach is key to the management of permanent maxillary central incisors affected by coronary anomalies.
			21	Mesiodistal	Ortho	
Smail-Faugeron V, 2016 (Case2) [37]		Male	21	Mesiodistal	Extraction	
Bulut H, 2017 [38]	To present the management of a fused maxillary central incisor with labial and palatal talon cusps that was moved through the midpalatal suture to obtain an appropriate dental midline by using a multidisciplinary approach.	Male	11	Mesiodistal	Ortho	A multidisciplinary approach, including orthodontics, periodontics, endodontics, and prosthodontics, could be used to achieve successful and satisfying treatment results.
Dorielo MCO, 2017 [39]	To describe a case of successful root canal treatment of an anomalous, fused inferior anterior mandibular incisor, using cone-beam computed tomography as a diagnostic aid.	Male	32	Mesiodistal	RCT	It confirms that anomalous teeth requiring root canal treatment pose many challenges to dental practitioners. In addition, it also reveals that new tools and materials are useful and can greatly improve treatment success.
Gera N, 2017 [40]	To present a rare case of triple teeth in permanent dentition in a 15-year-old female with associated periapical pathology.	Female	21	Mesiodistal	Extraction	Triple teeth also complicate endodontic, oral surgical procedures, and periodontal treatment apart from being unesthetic. Since the longitudinal grooves created by the fusion of three teeth are susceptible to caries, sealant therapy, and fluoride application may be necessary.
Persic Bukmir R, 2017 [41]	To report a conservative treatment of a rare developmental anomaly.	Male	11	Mesiodistal	RCT	It illustrates the importance of an individual approach when treating anomalous teeth. Priorities in pain and infection management to properly and functionally restore teeth should be unaffected by age.
Badole GP, 2018 [42]	To describe the endodontic management of upper central incisor fused with supernumerary root by using cone-beam computed tomography as a diagnostic aid.	Male	21	Mesiodistal	RCT	Cone-beam computed tomography helped in revealing complex tooth anatomy and the level of fusion of supernumerary teeth which was not visible in radiographs.
Kim EC, 2019 [43]	To present an eight-year-old patient with a permanent right central incisor that was fused with a supernumerary tooth as well as a geminated permanent left central incisor, and to describe the surgical-orthodontic-restorative management of the resultant malocclusion in the developing dentition.	Male	11	Mesiodistal	Partial extraction	It highlights physiological principles and demonstrates conservative, cost-effective, and clinically effective procedures in the management of developing malocclusions resulting from dental anomalies of fusion and gemination affecting permanent incisors.
Jarząbek A, 2020 [44]	To describe the multidisciplinary/minimally invasive treatment of fused immature permanent teeth.	Female	22	Mesiodistal	Partial extraction	The proposed multidisciplinary and minimally invasive treatment of the double tooth using a bioactive cement may facilitate the maturation of immature teeth and result in a desirable aesthetic and function.
Šarac Z, 2021 [45]	To describe a case of incomplete fusion of an unerupted mesiodens with a permanent maxillary central incisor, aligned in the dental arch.	Male	21	Mesiodistal	Partial extraction	A multidisciplinary collaboration is necessary for precise diagnosis and predictable treatment outcomes.
Sato M, 2021 [46]	To present the treatment of a fusion tooth by combining three-dimensional printing technology and endodontic intervention in a 10-year-old patient.	Female	12	Mesiodistal	Partial extraction and RCT	Techniques in modern endodontics, such as cone-beam computed tomographic imaging and three-dimensional printing, should be adapted when it is beneficial to patients.
Almutairi W, 2022 [47]	To present the clinical management of a fused mandibular third molar with a supernumerary tooth, wherein abnormal anatomy was ascertained using cone-beam computed tomography.	Male	48	Mesiodistal	Extraction	Successful treatment can be predicted when clinicians use a proper treatment plan and utilize all available diagnostic tools.
Present case		Male	12	Labiopalatal	Others	

## Data Availability

Data are contained within the article and Supplementary Materials.

## Supplementary Materials

The following supporting information can be downloaded at: https://www.mdpi.com/article/10.3390/children11010006/s1, Supplementary File S1: PRISMA 2020 Checklist.
